# Predictors of 30-day mortality in orthogeriatric fracture patients aged 85 years or above admitted from the emergency department

**DOI:** 10.1007/s00068-019-01278-z

**Published:** 2019-12-04

**Authors:** Henk Jan Schuijt, Jelle Bos, Diederik Pieter Johan Smeeing, Olivia Geraghty, Detlef van der Velde

**Affiliations:** 1 Department of Surgery, Sint Antonius Hospital, Nieuwegein, Postbus 2500, 3430 EM Utrecht, The Netherlands; 2 Department of Surgery, University Medical Center, Utrecht, The Netherlands; 3 Department of Internal Medicine and Geriatrics, Sint Antonius Hospital, Utrecht, The Netherlands

**Keywords:** Orthogeriatric, Traumatology, Fracture, Hip, Mortality, Geriatric

## Abstract

**Purpose:**

Orthogeriatric trauma patients are at risk for functional decline and mortality. It is important to identify high-risk patients in an early stage, to improve outcomes and make better informed treatment decisions. The aim of this study was to identify independent risk factors for 30-day mortality in patients aged 85 years or above admitted from the emergency department with a fracture.

**Methods:**

All orthopaedic trauma patients 85 years or above admitted from the emergency department were included. After a 30-day follow-up, mortality was determined by consulting the patient records. Multivariable logistics regression analysis generated odd ratios for mortality risk factors. A subgroup analysis was performed for patients undergoing hip fracture surgery.

**Results:**

The 30-day mortality in geriatric fracture patients admitted to the hospital was 12%. Risk factors for 30-day mortality were: increased age, male sex, decreased hemoglobin levels, living in an institutional care facility and a decreased BMI. For geriatric patients undergoing hip fracture surgery 30-day mortality was 11%. Independent risk factors for this group were: increased age, male sex, and a decreased BMI.

**Conclusion:**

Orthopaedic trauma patients aged 85 years or above who are admitted to the hospital with a fracture are at high risk for mortality. This study identified older age, male sex, and decreased BMI as predictors of 30-day mortality in admitted geriatric fracture patients and in geriatric hip fracture patients undergoing surgery.

## Introduction

Life expectancy is rising, and older orthopaedic trauma patients presenting to the emergency department (ED) are becoming a bigger part of the workload for orthopaedic surgeons [1]. Older patients often present with complex multidisciplinary medical problems, cognitive impairment and a higher level urgency, which complicates their evaluation and management [1]. Older orthogeriatric patients are also at risk for negative medical outcomes, such as functional decline and mortality [2]. It is important to identify high-risk patients in an early stage, in order to implement geriatric interventions to improve patient outcomes [3]. Identification of high-risk patients may also provide information for better informed treatment decisions and surgical management.

Patients aged 85 years or above constitute the fastest growing age group and are at even higher risk for postoperative complications and death than the general geriatric population [4–7]. These geriatric fracture patients are a distinct age group with considerable risk of negative medical outcomes. Many studies have been done that include these older patients, especially hip fracture patients. These studies have shown that age, male gender and comorbidity are important predictors of mortality, but few have specifically targeted the age group of patients aged 85 or above [6, 8]. Most studies focus on hip fractures and not the general population of geriatric orthopaedic trauma patients [8]. There is need for more research targeting this age group to identify risk factors for negative medical outcomes, which is why this study will exclusively target patients 85 years or above. The threshold of 85 years or above was chosen based on previous investigations [7, 9]

Additionally, this study will target the general geriatric population of fracture patients (i.e. any fracture regardless of treatment) as well as hip fracture patients undergoing surgery.

The primary aim of this study was to identify independent risk factors for 30-day mortality in patients 85 years or above admitted from the emergency department with any fracture. The secondary aim of this study was to identify independent risk factors for 30-day mortality in hip fracture patients aged 85 years or above undergoing surgery.

Methods.

### Study design and patient selection

The study period for this retrospective cohort study was 1-1-2012 until 31-12-2016. All patients 85 years or older presenting with a fracture at the ED who were admitted to the hospital were eligible for inclusion. Data collection was done by consulting the electronic patient files. This retrospective cohort study was conducted in a level 2 trauma center at St. Antonius Hospital, Utrecht, The Netherlands. The study was approved by the local institutional review board of St. Antonius Hospital and was performed in accordance with the ethical standards laid down in the 1964 Declaration of Helsinki and its later amendments. The Dutch Medical Research Involving Human Subjects act (WMO) did not apply to this study.

Identification of eligible patients was done using the diagnostic codes (DBC) for the most common fractures: wrist, fore arm, upper arm, shoulder, neck, vertebrate, pelvis, hip (proximal femur), distal femur, knee, lower leg and ankle. Patients were excluded if 1; primary survey was not performed at St Antonius hospital 2; if patients were discharged to another hospital or 3; if patients were admitted directly to intensive care unit 4; primary treatment was given at the ED, but the patient was not admitted 5; the patient had a pathological fracture or 6; the patient had a periprosthetic fracture. If a patient was admitted multiple times in the study period, only the first admittance was used.

### Measurements

A number of variables were collected based on literature and availability [6, 8, 10, 11]. The following pre-operative baseline variables were collected upon admission to the ED: age, sex, Body Mass Index/ Quetelet index (BMI), living situation prior to admission (at home, at home with home care, institutional care facility, other), whether or not the patient was living with a partner, number of different comorbidities (as mentioned in admission form), number of different medications, whether patients had experienced a previous episode of delirium, cognitive impairment (as mentioned in the admittance form, either declined or not declined), use of oral anticoagulants (yes/no), hemoglobin- (mmol/L), creatinine-(µmol/L), C-reactive protein (mg/L) levels. For patients undergoing surgery (regardless of fracture type) the following variables were collected: type of surgery (if any), type of anesthesia (general or regional, only applicable for patients undergoing surgery) and American Society of Anesthesiologists (ASA) classification (1 to 5).

### Outcome

The 30-day mortality was determined by consulting electronic patient files. For patients with an unknown date of death the last professional caregiver was contacted to ascertain the exact date of death.

### Statistical analysis

All statistical analyses were done using IBM SPSS Statistics for Windows, Version 25.0 (IBM Corp., 2017, Armonk, NY). The level of significance (*α*) was set at 0.05. Differences between deceased and surviving patients were analyzed at baseline. Normally distributed continuous data were presented as mean and standard deviation (SD) and tested with an unpaired *t* test. Not normally distributed continuous data were presented as median and interquartile range (IQR) and tested with a Mann–Whitney *U* test. Distribution was determined with the Shapiro–Wilk test for normality. All categorical and dichotomous data were tested with a chi-square test.

### Multivariable analysis

To reduce the number of possible predictors, candidate predictors to be included in the multivariable model were selected based on clinical relevance, availability, expert opinion and literature [12]. No univariable predictor selection was done which is in line with current recommendations by expert in the field of prediction modelling as it introduces data driven predictor selection bias [12, 13]. A full model approach was used, with at least 10 events per variable [14]. Missing data in the initial cohort were analyzed for patterns using Little’s missing completely at random (MCAR) test except for ASA classification and type of anesthesia, which were missing for all patients who did not undergo surgery. Data missing completely at random (MCAR) were imputed using multiple imputation techniques (5 imputations).

### Subgroup analysis

Because hip fractures are the most common indication for surgery in orthogeriatric trauma patients, a subgroup analysis was performed for all hip fracture patients undergoing surgery. Missing data for all variables including ASA classification and type of anesthesia were analyzed for patterns using Little’s MCAR test. An additional multivariable logistic regression analysis was performed to calculate the odds ratio (OR) for the selected candidate predictors in this subgroup.

## Results

### Baseline characteristics

In total, 810 eligible cases were identified, 83 of which met the exclusion criteria and 35 patients were admitted two times during the study period. This resulted in an included cohort of 692 patients. After 30 days a total of 86 patients (12%) had deceased. Baseline characteristics of survivors and deceased patients are summarized in Table 1.

**Table 1:** Baseline characteristics of 30-day mortality vs. survivors. All percentages are calculated for valid data (i.e. excluding missing data)

Characteristics	Total (*n *= 692)	Missing	30-day mortality (*n *= 86)	Survivors (*n * = 606)	*p* value
Age, median (IQR)	89 (87–92)	0	90.5 (87–94)	89 (87–92)	** < 0.01**
Male sex, *n * (%)	149 (22%)	0	29 (34%)	120 (20%)	** < 0.01**
BMI (kg/m^2^), median (IQR)	24 (21–26)	180	21 (19–24)	24 (22–26)	** < 0.01**
Living situation, *n * (%)		27			** < 0.01**
At home / at home with care	350 (53%)	36	29 (35%)	321 (56%)	** < 0.01**
Living in institutional care facility	306 (47%)	36	54 (64%)	252 (44%)	** < 0.01**
Living with partner, *n * (%)	107 (16%)	17	16 (19%)	91 (15%)	0.39
Comorbidity					
Number of comorbidities, median (IQR)	3 (2–5)	62	4 (2–5)	3 (2–5)	** < 0.01**
Number of different medications, median (IQR)	6 (4–8)	69	7 (5–10)	6 (4–8)	** < 0.01**
Prior delirium, n (%)	199 (31%)	40	35 (44%)	164 (29%)	** < 0.01**
Impaired cognitive functioning, *n * (%)	278 (42%)	29	47 (57%)	231 (40%)	** < 0.01**
Use of oral anticoagulants, *n * (%)	392 (62%)	63	61 (78%)	331 (60%)	** < 0.01**
Biomarkers					
Hemoglobin (mmol/L), mean (SD)	7.5 (1.0)	88	7.2 (1.1)	7.6 (1.0)	** < 0.01**
Creatinine (µmol/L), median (IQR)	79 (64–100)	195	95 (74–109)	78 (63–98)	** < 0.01**
C-reactive protein (mg/L), median (IQR)	5 (1–18)	153	6 (1–31)	5 (1–18)	0.56
Type of surgery, n (%)		5			0.15
Spinal column	2 (0%)		1 (1%)	1 (0%)	
Proximal humerus	11 (2%)		3 (4%)	8 (1%)	
Distal humerus	2 (0%)		0 (0%)	2 (0%)	
Hip fracture (proximal femur or collum)	492 (72%)		55 (65%)	437 (73%)	
Distal femur	18 (3%)		3 (4%)	15 (3%)	
Ankle	19 (3%)		0 (0%)	19 (3%)	
Other trauma surgical procedure	1 (0%)		0 (0%)	1 (0%)	
Conservative treatment	142 (21%)		23 (27%)	119 (20%)	
Type of anesthesia, *n * (%)^a^		13			0.57
General	452 (84%)		52 (12%)	400 (89%)	
Regional	85 (16%)		8 (9%)	77 (91%)	
ASA classification, *n * (%)^a^		81			** < 0.01**
1	14 (3%)		0 (0%)	14 (3%)	
2	217 (46%)		17 (30%)	200 (48%)	
3	230 (49%)		34 (62%)	196 (47%)	
4	8 (2%)		4 (7%)	4 (1%)	
5	0 (0%)		0 (0%)	0 (0%)	

^a^Percentages and missing data calculated for patients undergoing surgery

Significant differences presented in bold

Patients who died during follow-up were older at baseline than survivors. Deceased patients were more often male, and they had a lower BMI. Patients living in an institutional care facility were more likely to die during follow-up. Patients who died had more comorbidities than survivors and used more medications. A previous episode of delirium was associated with 30-day mortality, as was impaired cognitive function. The use of oral anticoagulants was higher in the deceased group, hemoglobin levels (mmol/L) were lower, and creatinine levels were higher. A higher ASA classification was associated with 30-day mortality.

### Missing data and multivariable analysis

In the initial cohort, missing data were missing completely at random (Little’s MCAR test *p* = 0.702). In the subgroup, all missing data, including ASA classification and type of anesthesia, were also missing completely at random (*p* = 0.625). The results of the multivariable analysis are shown in Table 2. It showed that age was an independent risk factor for 30-day mortality (OR 1.11 for each year above 85 years), as was male sex (OR 2.96) and living in an institutional care facility (OR 2.31). Each 1 mmol/L decrease in hemoglobin increased the chance of mortality (OR 1.34), as did each 1-point decrease in BMI (OR 1.15). Previous episodes of delirium, the use of oral anticoagulants or surgical intervention were not independent predictors of mortality in this study.

**Table 2:** Multivariable analysis for all admitted patients and subgroup analysis for all hip fracture patients undergoing surgery

Initial cohort (*n * = 692)	Adjusted OR	95% CI	*p* value
Age (per year above 85)	1.11	1.04–1.18	** < 0.01**
Male sex	2.96	1.68–5.23	** < 0.01**
Living in an institutional care facility	2.31	1.34–3.99	** < 0.01**
Previous episode of delirium	1.32	0.76–2.30	0.32
Hemoglobin (each 1 mmol/L decrease)	1.34	1.06–1.70	**0.02**
BMI (each point decrease)	1.15	1.02–1.29	**0.02**
Use of oral anticoagulants	2.22	0.88–5.56	0.09
Surgical intervention for any fracture	0.60	0.34–1.06	0.08
Hip fracture patients undergoing surgery (n = 492)			
Age (per year above 85)	1.14	1.05–1.25	** < 0.01**
Male sex	3.09	1.56–6.10	** < 0.01**
Living in an institutional care facility	1.94	0.99–3.79	0.05
Hemoglobin (each 1 mmol/L decrease)	1.25	0.93–1.70	0.14
BMI (each point decrease)	1.22	1.02–1.46	**0.03**
ASA classification (per class increase)	1.93	0.97–3.83	0.06

Significant differences presented in bold

The subgroup analysis for patients with hip fractures undergoing surgery consisted of 492 patients, of whom 55 died during follow-up (11%). The multivariable analysis for this group showed similar results for age (OR 1.14 for each year above 85 years) male sex (OR 3.09) and BMI (OR 1.22) as independent predictors of mortality. ASA classification and living in an institutional care facility were borderline significant. Hemoglobin levels at presentation at the ED were not a statistically significant independent predictor of mortality in this subgroup.

## Discussion

### Red line and take-home message

This study shows that 30-day mortality in geriatric patients admitted to the hospital with a fracture is high, regardless of treatment (12%). There are several independent risk factors for 30-day mortality in this population: increased age, male sex, decreased hemoglobin levels, living in an institutional care facility and a decreased BMI. For geriatric patients undergoing hip fracture surgery, 30-day mortality was 11%. Independent risk factors for this group were: increased age, male sex and decreased BMI.

### Comparison with previous studies

Previous studies investigating risk factors for mortality in geriatric fracture patients have targeted patients aged 65 or above. In one such study, age was found to be a risk factor for mortality, which corresponds with our results. Higher injury severity and low systolic blood pressure were also found to be predictors of mortality in younger cohorts, but detailed information on blood pressure and severity of injury per body region were unavailable in our cohort [15].

Predictors of mortality in older hip fracture patients have been extensively studied but not in the patient group aged 85 years or above [8, 16]. Age was found to be an independent predictor of mortality in both these studies but was dichotomized in age groups below 85 years or 85 years and above. Hemoglobin level and ASA classification were also independent predictors but were analyzed as dichotomous outcomes [9, 10]. Dichotomization results in loss of information and predictive power [17]. In this study, age, hemoglobin was analyzed as a continuous outcome and ASA classification as a categorical variable to address this problem.

Male sex was found by previous studies to be a risk factor for 30-day mortality in hip fracture patients with an OR of 1.66 (95% CI 1.15–2.39) [9]. In this study, an OR of 3.09 (95% CI 1.56–6.10) was found, suggesting that male orthogeriatric trauma patients aged 85 years or above are at even higher risk of mortality. This would confirm that risk factors in geriatric fracture patients aged 85 years or above are distinctly different from their younger counterparts.

### Strengths and limitations

This was the first study to investigate risk factors for 30-day mortality in general geriatric fracture patients and hip fracture patients aged 85 years or above. The cohort was very large and there was no loss to follow-up. Another strength is the analysis of continuous outcomes without dichotomization, unlike previous studies [9, 10]. In this study, predictor selection bias was reduced because there was no data-driven predictor selection. Because the cohort consisted mainly of hip fracture patients (71%), a subgroup analysis was performed to correct for this.

This study has a few limitations. Only patients admitted to the hospital from the ED were included. This means that patients who were treated and discharged from the ED were not included, which leads to possible selection bias. Very few studies include these patients because follow-up data of these patients are often unavailable. This selective follow-up is a challenge in geriatric trauma research but can be addressed by searching death registries or telephone follow-up [18]. The authors of this study recommend that these patients are included in future investigations, to get a more accurate representation of the ED population. Another limitation is the amount of data missing at baseline. This is inevitable in retrospective cohort studies, but it also reflects that different caregivers collect and record different patient characteristics. This illustrates the need for more standardized management of these patients and the relevance of this study.

### Interpretation of results

Almost all hip fracture patients are admitted directly from the ED, while patients with other fracture types are not always admitted. This means that patients with a fracture other than a hip fracture are likely to have a worse prognosis at baseline due to overrepresentation of relatively healthy hip fracture patients. Geriatric hip fracture patients are indeed notorious for adverse medical outcomes [19]. It is important to realize that hip fracture patients who received conservative treatment were not included in the subgroup analysis. The number of hip fracture patients in the conservative treatment group was negligible, but mortality in this group was high nonetheless (16%). It is likely that patients who received conservative treatment were patients with a poor prognosis.

A decreased BMI was found to be a risk factor in both the total cohort and hip fracture surgery subgroup. These results should be interpreted with some caution, as there was a lot of missing data for this variable (*n* = 180). Previous high-quality studies in hip fracture patients have not found a relation between BMI and mortality, although these studies did not specifically target patients aged 85 or older [6, 16].

It is important to realize that the BMI might not be the best parameter for nutritional status. Patients with a high BMI may still be malnourished. In future research, scoring systems, such as the short nutritional assessment questionnaire (SNAQ) or malnutrition universal screening tool (MUST) [20, 21], should be investigated as screening methods for 30-day mortality in orthogeriatric trauma patients.

Living in an institutional care facility (*p* = 0.05*)* and ASA classification (*p* = 0.06*)* were borderline significant predictors of 30-day mortality in hip fracture patients undergoing surgery. This is likely the results of a small sample size for the subgroup analysis. ASA classification has been shown to be a predictor of 30-day mortality in non-geriatric hip fracture patients in previous studies [22, 23]. Living in an institutional care facility has also been shown to be a risk factor for both patients aged 70 years or above admitted from the ED in several studies [24, 25] and in hip fracture patients presenting at the ED [9, 10]. The number of events per variable in the hip fracture subgroup analysis was 9.2 which is slightly lower than the commonly used 10 events per variable in this type of analysis [12]. There is no scientific evidence that the number of events per variable should be at least 10, and simulation studies have shown that an event per variable rate between 5 and 10 can be acceptable in most cases [13, 26]. Nevertheless, it may still indicate that the sample may have been too small to detect a significant difference between deceased patients and survivors in this sample. Therefore, both these variables cannot be ruled out as predictors of 30-day mortality and merit further investigation.

During the study period, there was no integrated orthogeriatric care unit in St. Antonius hospital. Orthogeriatric care units have been shown to improve patient outcomes [27, 28]. By identifying patients at risk for negative medical outcomes, geriatric interventions can be targeted at those patients that would benefit from them. However, there was a geriatric awareness program which increased awareness for the most common complications during admission for these patients.

### Clinical relevance

This is one of the first studies to investigate geriatric fracture patients in the age group of 85 years and above. Very little is known about this rapidly growing group of patients who are at much higher risk of negative medical outcomes than younger patients [4, 5]. There is urgent need for more research into screening methods and medical outcomes in very old geriatric fracture patients.

## Conclusion

This study shows that all older orthogeriatric trauma patients who are admitted to the hospital with a fracture have a high risk (12%) of 30-day mortality, regardless of treatment. Several routinely collected predictors of 30-day mortality in admitted geriatric fracture patients were identified. In the population of geriatric fracture patients, independent risk factors for mortality were: increased age, male sex, living in an institutional care facility, decreased hemoglobin levels or decreased BMI. For geriatric hip fracture patients, independent risk factors were: increased age, male sex and decreased BMI. The authors advocate to regard any fracture patient aged 85 or above as a high-risk patient.
